# Reconsidering nutrition science: critical reflection with a cultural lens

**DOI:** 10.1186/1475-2891-13-42

**Published:** 2014-05-02

**Authors:** Craig A Hassel

**Affiliations:** 1 Department of Food Science & Nutrition, University of Minnesota, 1334 Eckles Avenue, St. Paul, MN 55108, USA

**Keywords:** Nutrition science, Philosophy, Hidden subjectivity, Culture, Epistemology, Cross-cultural engagement

## Abstract

**Background:**

European culture gave birth to modern science as a means to investigate and explain the natural world. The biomedical disciplines that have since emerged, including nutrition, presuppose a web of basic presuppositions, background assumptions and implicit cultural values that are often overlooked and escape peer review. These "hidden subjectivities" are widely taken-for-granted while exerting a powerful hold on the scope, direction and patterns of disciplinary thought. Nutrition science currently has no accepted means of collectively attending to hidden subjectivities embedded within its methods and practice. Here I propose that directing inquiry into these dimensions holds potential to advance our discipline.

**Methods:**

This critically reflective approach emerged from critical theory and the practice of cross-cultural engagement (CCE). CCE deliberately seeks out and critically engages food and health understandings of non-European cultures. Its protocol includes cognitive frameshifting, a practice of temporarily stepping outside of habitual thought patterns and into a non-biomedical framework of background assumptions. A cultural lens metaphor derives from CCE practice and is forwarded here as a viable means for restoring critically reflective attention to hidden subjectivities while also inviting further CCE practice within the discipline.

**Results:**

Critical reflection with a cultural lens allows cognitive attachments to materialism, reductionism, mechanistic thought, naïve realism, control over nature and pervasive subject-object dichotomies between mind and matter, scientist and nature, experience and reality, among many others to become more available for critical consideration. Culturally diverse food and health understandings otherwise dismissed as "unscientific" or held in abeyance gain value as alternative assumptive frameworks and cognitive models that can be temporarily inhabited for further intercultural reflection and insight.

**Conclusion:**

Critical reflection with a cultural lens allows reconsideration of nutrition science in light of its culturally specific origin and foundation. This perspective can advance the discipline in two ways. First, it extends skeptical inquiry into hidden subjectivities that are otherwise implicit and seldom given over to critical consideration. Second, it can broaden scholarly inquiry through deliberate attempts to cross cognitive boundaries and empathically inhabit different cognitive worlds. This developmental practice holds potential to both deepen and broaden disciplinary inquiry.

## Introduction

### Geissen, von Liebig and History

In 2005, a gathering of nutrition professionals organized by the International Union of Nutritional Sciences and the World Health Policy Forum assembled at the University of Giessen to consider how the nutrition science and professions could best meet the future challenges that the 21^st^ century would bring. Out of this gathering emerged "The Giessen Declaration" [1], which begins as follows:

"Now is the time for the science of nutrition, with its application in food and nutrition policy, to be given a broader definition, additional dimensions and relevant principles, to meet the challenges and opportunities faced by humankind in the twenty-first century………. The food, agriculture and nutrition sciences were originally devised in the midnineteenth century in Europe, notably by Justus von Liebig at the University of Giessen in Germany, where our meeting has been held".

Those assembled at Giessen justified their 2005 efforts to consider a "new nutrition science" in part because "the world in which we now live is very different from the world in which nutrition as a science was framed" [1]. We need only look back over the past 50 years to see a significant shift from questions of acute deficiency diseases to the much more complex and systemic problems of diet-related chronic disease. Looking ahead, many of our students are now questioning whether the discipline of nutrition science is equipped to deal with concepts of human well-being seen as something independent of pathology or beyond the biomarkers of disease prevention. Given a rapidly changing world, it becomes important to ask how our discipline of nutrition science should respond to the changing times. What is the meaning of a "new nutrition science"? What exactly is meant by "a broader definition, additional dimensions and relevant principles"? What about nutrition science should change and what should not? How ought we determine the implications and think through proposed changes?

While some of these questions were addressed in 2005 [2], I suggest that they are the kinds of questions we should not solve too hastily. These are the kinds of questions that require continuing deliberation and thoughtful discourse even though they are of a somewhat different nature than those emerging within everyday nutrition research. Such questions point us toward a realm of philosophy and epistemology, a realm that our discipline seems to have moved away from over the past century yet one that continues to sit quietly, almost subconsciously beneath the ongoing practice of nutrition science. This is what most scientists tend to think *with*, not *about*. I believe we have come to a point in time where nutrition science would be well-served in re-directing scholarly attention toward this intangible realm, one usually associated with philosophy of science [3-6]. A brief glimpse of history may be helpful in illuminating what is often implicit.

The elements of modern scientific practice can be traced to ancient Greece, but many point to the significant impact of Sir Francis Bacon and his 1620 publication Novum Organum [7] for laying out the groundwork of scientific principles and practice. Although Bacon used the word science, the work he described was then the practice of *natural philosophy* and the work was written to his peer group of *natural philosophers*. Specific use of the word *science* in a professional context is by comparison, relatively recent [8-10]. In 1831, about the time von Liebig was studying foods in his chemistry laboratory at Giessen, the word science was first widely employed as we understand it today, replacing the term natural philosophy [8]*.* Science was a word deliberately selected by a new social institution, the British Association for the Advancement of Science (BAAS), for purposes of professionalization [8-10]. The founders of BAAS were seeking a label of professional distinction that would set their work apart from natural philosophers, from technologists steeped in the successes of the Industrial Revolution, and from that of the Royal Society [9]. In archaic English, *science* simply meant knowledge (Latin: *scientia*) but in the context of its BAAS origin, science would narrowly privilege an operational meaning defined, in part, by the approaches to the forms of inquiry then being taught in universities [10]. It was used by BAAS to create a position from which to lobby financial support for its members and to distinguish the emerging university curricula [9]. The BAAS use of the term served as a model for the American Association for the Advancement of Science in 1848.

These newly proclaimed scientific professions were steeped in the Newtonian-Cartesian worldview of the day. This view of the world emerged out of the Copernican revolution and the subsequent scientific revolution and European Enlightenment [11]. In it, the physical world is seen as an atomistic system of objects governed by mechanistic, mathematical laws. Such laws are comprehended and described through careful observation, autonomous reason and analytical thought. An atomistic world demanded a specific type of interpretation calling for concrete prediction of its mechanistic, structural, material and impersonal nature. It called for carefully controlled empirical observation in the manner articulated by Bacon. It called for skepticism and a disciplined, critical rationality from professionals who went to great lengths to construct ideal conditions for rigorously controlled observation and experiment. It called for reductionist inquiry, a form of inquiry that von Liebig and other chemists would employ with great success in revealing fundamental chemical and material composition of nature, beyond mere appearance [11]. These dynamics also facilitated the development of increasingly specialized and focused disciplines and sub-disciplines. Scientists working within these environments would increasingly come to understand their practice as operating apart from political and societal concerns to the extent possible [11,12]. This worldview also grounded the German idea of a university as a place to do research that contributes to fundamental knowledge through the practice of science [13]. This German idea was imported to American universities and first took root in the 1870’s at Johns Hopkins University, quickly spreading to other universities throughout the US including Michigan, Columbia and Chicago, among others [13].

Needless to say these newly established professions and institutionalized approaches to research met with tremendous success. By the early 20^th^ century, the trajectory of knowledge advancement had earned the scientific professions a distinguished societal position to deliver authoritative judgments concerning questions of fact and truth [12]. Aligned with their training and in accordance with a Cartesian mindset, scientific professionals positioned themselves as detached spectators, separate and distinct from the material objects of their study [11-13]. Social, cultural and other circumstances and interests lying outside the domain of observation and controlled experiment were deemed epistemologically irrelevant [11,12]. Only data from experiment and observational measurement would determine the acceptance or rejection of propositions in the quest for objective knowledge of the world [12]. Third-person empirical inquiry and professional peer critique were invoked to separate discovery from the context of justification as a means to validate scientific work, further guarding it from subjective bias or outside influence [13]. The 20^th^ century work of understanding and ameliorating acute deficiency diseases offers us an excellent example of the success of this scientific approach when applied to problems of a deterministic, cause/effect nature.

With the success of the growing professions, scientific practice became further distanced from its roots in natural philosophy. Over time, philosophy of science became less and less an integral source of meaningful guidance for scientific practice within the rapidly proliferating professional societies [11,12]. This legacy is exemplified in that many scientists practicing today have not been required to formally study philosophy or history of science. In the context of increasingly narrow and specialized disciplinary inquiry, practicing scientists would come to see their work more as about describing reality directly and less as about constructing coherent mental maps derived from a particular method of questioning reality [11]. Scientists would emphasize the more tangible dimensions of their work as direct observation and testing in carefully controlled conditions so as to exclude the influence of human subjectivity to the extent possible [12]. Metaphysical and philosophical consideration for background assumptions carried into the formulation of hypotheses became ancillary or disregarded altogether [3,11].

Success of scientific professions in providing benefits to society would confer back to them much subsequent authority to define nature and to determine what constitutes reliable knowledge of the world [11]. This authority, combined with continuing disengagement from philosophical and social perspectives would serve to reify a belief among scientists that *legitimate human knowledge can only arise* through methods accepted as valid within scientific societies [12]. People in society not academically qualified or engaged in scientific research were not expected to provide cogent, authoritative criticisms of scientific results arising out of their own personal experience [12]. The ideas or thoughts of non-professionals could be studied empirically but would not in themselves constitute serious contributions to scientific, academic knowledge. Although it has always been characteristic of humans to create practical knowledge about the world – whether experiential, tacit know-how, empirical knowledge or ancestral knowledge passed down through generations – these along with other sources of human knowledge created outside the wake of professional scientific advancements were either held in abeyance by the scientists until they could be appropriately tested or summarily dismissed [14-17]. Professional authority over knowledge further encouraged a vision of science as directly describing the realities of the natural world, beyond mere appearance. Thus, fertile ground was created for reification of implicit background assumptions and basic presuppositions [12,18]. Let us turn our attention for a moment to this important realm of background assumptions.

### Hidden subjectivity

About 50 years ago, as nutrition scientists began shifting attention from acute deficiency diseases toward complex diet-related chronic diseases, Thomas Kuhn coined the term "paradigm" in referring to the shared models of how the world works as well as the shared understanding of rules and standards of scientific practice that prepares the student for membership in a particular scientific community [4]. Kuhn described the mental models supporting a paradigm as important because they exert a deep hold on the scientific mind; a powerful influence to think of and perceive issues in one way rather than another [4]. These shared understandings of a scientific community allow certain categories and relationships to emerge as especially salient (bioactive molecules), while others become less noticeable or invisible (one’s relationship with food). The epistemologist Lorraine Code coined the term "hidden subjectivity" in referring to the background assumptions and hypotheses implicit to and embedded within a well-established scientific discipline [5]. The term hidden subjectivity points to the subjective nature of background assumptions and implicit hypotheses. Materialism, reductionism and mechanistic thought for example, are not filtered out by objective, disinterested techniques but represent subjective orientations that permeate the mental models through which disciplinary inquiry proceeds [3,6]. Subjective orientations are hidden to the extent they escape peer-review and skeptical inquiry. When background assumptions are shared by all members of a scientific community they acquire an invisibility that renders them unavailable for criticism [6]. Hidden subjectivities present a problem of epistemology, one that philosophers of science refer to as underdetermination – gaps between hypotheses and data - when background hypotheses are not articulated but presupposed as universal givens [6].

"Hidden subjectivity" is a handle I will use in referring to the realm of scientific thought that is often implicit to practicing scientists and therefore easily overlooked. This realm is hidden in part because since the time of von Liebig, the life sciences, including nutrition science have grown detached from their roots in natural philosophy. Matters of philosophy and epistemology are no longer central concerns in advancing nutrition science. Success in science also plays a role. As Kuhn observed, implicit understandings greatly facilitate scientific advancement because it frees scientists from the work of consciously attending to implicit mental models and shared understandings so that they can direct full attention toward solving the many nuanced puzzles left within the theoretical framework of the discipline [4]. Over time and with success, these hidden subjectivities offer a scientific community a great sense of strength and identity even as they become subconsciously embedded within the disciplinary mindset [3,6]. Precisely because mental models are successful, over time the web of shared presuppositions supporting them become unconsciously taken for granted [3-6]. They become invisible and thus are easily overlooked by practicing scientists [3]. Ludwig Fleck, a microbiologist by training, observed that once a structurally complete system of beliefs consisting of many details and relations has been formed (meinungssystem), it becomes closed, circular and offers tenacious resistance to anything that contradicts it [19]. The realm of hidden subjectivity is important because these ideas hold significant power in governing thought within a scientific community. It follows that explicit discussion of implicit mental models and taken-for-granted presuppositions offers both opportunities and challenges for a scientific discipline.

### Hidden subjectivity and nutrition science

In nutrition science, the hidden subjectivities that proved so successful in ameliorating acute deficiency diseases were re-directed largely intact toward diet-related chronic diseases of a quite different nature. Is it possible that over-attachment to a scientific mindset that proved so successful with acute deficiency diseases might in some ways actually represent an obstacle to success as our attention has shifted to the newer challenges of a more complex, systemic and chronic nature? I believe this question deserves explicit and careful consideration.

**Figure 1:**
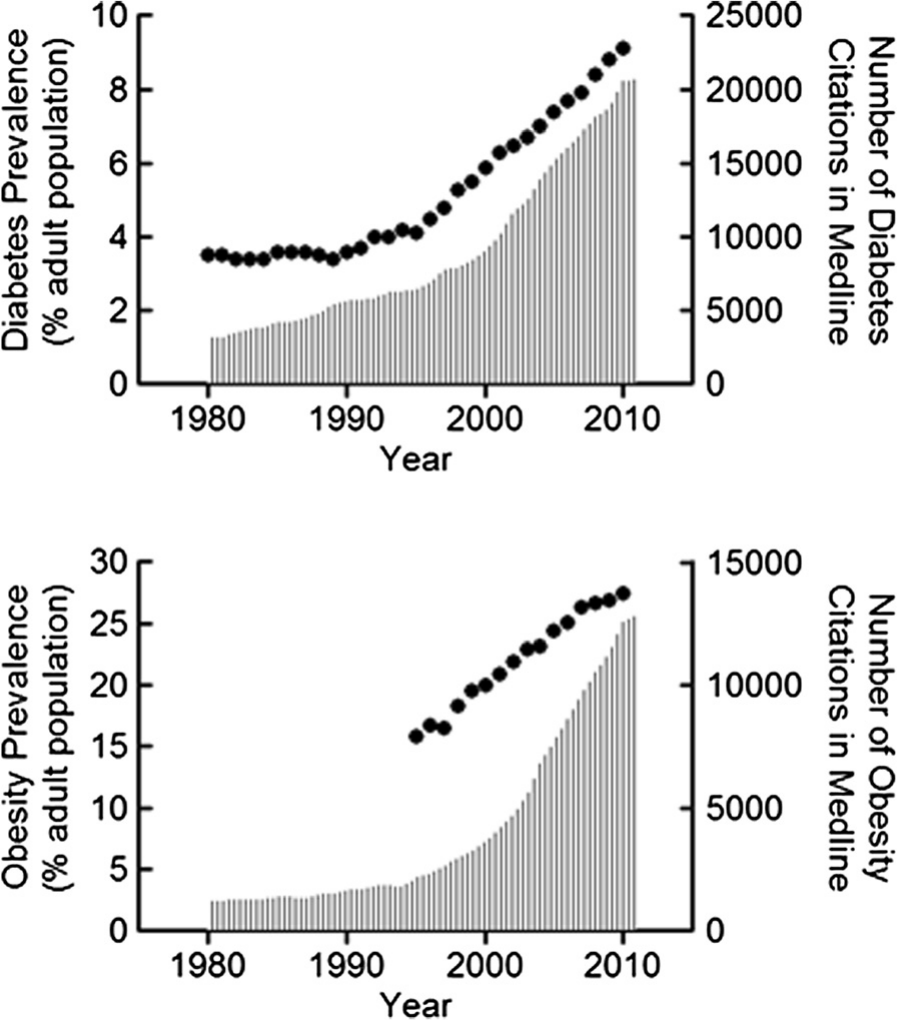
Yearly population prevalence of diabetes (upper panel) or obesity (lower panel) plotted alongside growth in the fund of professional knowledge as indicated through number of publications brought up in a corresponding keyword search in the Medline database.

Consider the relationship between our fund of accumulating academic knowledge for either obesity or diabetes as plotted against corresponding disease incidence rates (Figure 1). Why does our wealth of academic knowledge not translate more directly to improving the human condition? I understand that many factors play into chronic disease incidence that go well beyond the domain of nutrition science. My point in showing Figure 1 is to ask whether our hidden subjectivities might also have a role in this relationship. Recent calls of over-attachment to a reductionist, pharmacologic mindset when considering the complex food matrix [20,21] point to difficulties with hidden subjectivities that manifest as habits of mind seeking linear, deterministic relationships, as one example. Frustrations with attempts to create relevant dietary guidance from the most rigorous and systematic reviews of scientific research [22,23] are grounded in the "hierarchy of evidence" guiding such review [23]. The hierarchy gives greatest weight to certain kinds of evidence/methodologies (randomized controlled trials or RCT) [23]. Is this hierarchy an expression of unexamined hidden subjectivities? Is this weighting of evidence toward internal validity truly the most appropriate choice for the work of addressing the highly complex and systemic matters of diet and health relationships? What about systems thinking [24] as a tool? And how is the discipline of nutrition science equipped to deal with concepts of human well-being seen as something independent of pathology or beyond the biomarkers of disease prevention? These important questions concern epistemology and hidden subjectivity. Is it possible that directing inquiry into these dimensions might help advance nutrition science?

## Methods

This approach to reconsidering nutrition science stems from participatory action inquiry [25] grounded in critical theory [26], critical thinking [27] and an emerging practice of cross-cultural engagement (CCE) [28-30]. CCE is a practice relevant to nutrition science because every society has had to develop its own understandings of food and health relationships [31,32]. Older, non-European knowledge traditions such as indigenous knowledges [14,17,32], Ayurveda [33] and Chinese medicine [16,34] tend to be summarily dismissed as untenable because underlying concepts and theories such as Qi, yin/yang theory and dosha theory seem incomprehensible or even absurd when considered from within a biomedical perspective [16,28]. While such a reaction is certainly understandable, it can also foreclose the opportunity for further critical consideration and potential insight. By contrast, a CCE approach would ask nutrition professionals to think again. Its practice is one of actively seeking out well-established food and health understandings of non-European cultures precisely because they originated beyond the scope and *a priori* of biomedical science. Non-biomedical understandings are approached as perspectives that can be temporarily inhabited for novel cognitive vantage points from which to consider diet and health issues [28,30]. CCE practice holds that what might initially seem absurd or incomprehensible is likely a superficial manifestation of deeper cultural difference playing out at the level of background assumptions and mental models. Does cultural difference in itself pose sufficient rationale to prohibit further consideration of non-biomedical understandings by nutrition professionals?

CCE offers a critically reflective and developmental practice through which to explore foreign cognitive terrain and to navigate the attendant cognitive dissonance that often accompanies this work [28-30]. It first asks the nutrition professional to reconsider the idea that familiar biomedical models represent universal descriptions of reality. CCE asks scientists to consider biomedical research as constructing mental models or as building elaborate maps derived from a particular (biomedical) approach to questioning reality. Such an orientation fits well with the idea of model-dependent realism [35]. Model-dependent realism is based on the understanding that all perception of the world, including scientific observation, occurs through pre-existing interpretive filters, mental models of human construction. This allows for the latitude of useful yet very different mental models regarding the same physical phenomena or situation (for example, classical Newtonian physics and quantum mechanics) [35]. It is not surprising that the human species has created a rich diversity of non-biomedical mental models for understanding diet and health relationships [31,32,36,37]. CCE emphasizes the cultural dimensions of different mental models for further consideration and exploration.

Second, a CCE approach asks the nutrition professional to temporarily suspend initial questions regarding validity. This allows for the additional latitude needed so that a non-biomedical perspective can be inhabited cognitively and experientially, creating the possibility for it to be explored from within from within its original cognitive frame of reference. This includes stepping into and experiencing its different fabric of background assumptions and cultural subjectivities that constitute the mental model and its view of diet and health relationships [28-30]. Navigating cultural difference at these deeper dimensions can evoke a disquieting sense of cognitive dissonance. Part of CCE practice includes developing a capacity to tolerate dissonance, resisting the impulse to resolve or dispose it in favor of holding the questions for further contemplation [28]. Cognitive dissonance is discussed further in the section below entitled "The Healthful Dissonance of a Cultural Lens".

Cognitive frameshifting [38] is a developmental ability and an advanced intercultural skill that may be unfamiliar to many nutrition professionals [28]. It is a deliberate practice of seeking and engaging cultural difference as an opportunity to temporarily inhabit a different worldview orientation, to cognitively step into its background assumptions and mental models to the extent possible [28,38]. CCE applies the practice of cognitive frameshifting to culturally different understandings of food and health relationships. Its successful practice requires some significant appreciation for and sensitivity to the complexity and nuance of the less tangible, yet powerful governing dimensions of culture [28,30,36,38]. A basic protocol for this critically reflective practice comes from almost 20 years of experience and can be outlined as follows:

1. Develop ongoing and personal relationships with individuals who work within non-biomedical perspectives.

2. Maintain an open-minded disposition by adopting the flexibility afforded by model-dependent realism and by suspending any impulse toward preliminary judgments regarding validity or tenability of foreign concepts.

3. Begin to recognize and reflect upon the cultural nature of your own habitual thought patterns and mental models,

4. Begin to recognize and reflect upon your own cognitive attachments to your habitual thought patterns and mental models.

5. Develop a capacity to temporarily loosen your attachment to thought patterns and mental models.

6. Experientially and episodically dwell for brief periods of time within the context of non-biomedical background assumptions and mental models [28-30,36], thereby developing cognitive frameshifting capacity [38].

7. Critically reflect on your experience with each of these steps.

This protocol outlines a developmental practice that makes it possible for professionals to temporarily step out of their scientific mindset and inhabit unfamiliar mental models. This unfamiliar territory offers different cognitive locations from which to: 1) critically reflect back upon and observe one’s scientific mind, its hidden subjectivities and its cultural tendencies in the practice of nutrition science; and 2) consider unfamiliar mental models from within their own cognitive frame of reference [28]. I refer to this critically self-reflective orientation metaphorically as a cultural lens. In the next section, I begin with a few insights from my own experience for the benefit of those who might have further interest in taking up CCE. I then attempt to build a case that the self-reflective orientation afforded by the cultural lens metaphor deserves a place within the broader nutrition discipline.

## Results and discussion

For readers curious about the CCE experience, I offer a few cursory insights and observations from my own experience. It is important to emphasize here that I am not suggesting that all nutrition professionals develop a practice of CCE. My argument is that the collective discipline itself would benefit from even a a small cadre of CCE practitioners within its ranks who would then be positioned to share their emerging insights in discourse with the greater collective.

Although CCE may strike readers initially as perhaps overstepping the boundaries of credible scientific practice, it does not ask that I abandon my scientific commitments or compromise my scientific integrity. I need not uncritically accept other cultural knowledges or adopt a cultural heritage other than my own. Quite the contrary, through critical reflection and contemplation of hidden subjectivities I become more consciously aware of the cultural dimensions of scientific practice, better grounded culturally and more informed philosophically. I am able to bring critical thinking, critical self-reflection [26,27] and intellectual humility [37] to better understand and embrace subjective cultural dimensions of knowledge that may not have been previously apparent [28]. CCE feels somewhat different than more instrumental forms of disciplinary inquiry because it allows for a transformative dimension. Transformational learning involves not only changes in *what* we know (informational learning), but also changes in *how* we know [39]. I am more able to open my full self to experiencing subjective realms of cultural difference without becoming defensive or retreating to a place where I am left only to assert positional power or the intellectual authority inherent in my credentials or position [28,37].

I also find that personal relationships with non-academic mentors characterized by mutual trust and respect are essential in learning how to respectfully engage and navigate cultural difference without compromising human dignity or scientific integrity [28-30,37]. I become more self-aware of the impulse to frame any problem or understand any issue through the lens of my academic training, and become more sensitive over time to how reflexively imposing this lens can distort knowledge that is generated from different cultural orientations [14,16,28-30,36]. I become better prepared to recognize the power differentials and cultural assimilation that are built into institutional structures of higher education, yet often invisible to those within. Issues of injustice become more visible as I learn to navigate the sometimes conflicting and unsettling terrain of cultural difference [28-30,36,37]. Such issues are prominent within the narratives of many cultural communities [14,16,17,36,37] but are rare within disciplinary discourse [30]. The ability to see problems from different cultural vantage points while respecting the value of these perspectives is a practice that can seem messy and complicated, but also one that I believe holds significant potential for innovation [30,37]. As a developmental craft, the practice of CCE includes skills increasingly demanded by a rapidly changing and diverse society that now awaits our students [18,28,36].

### The healthful dissonance of a cultural lens

From the very brief historical sketch above, it should be obvious that today’s nutrition science is of Eurocentric origin. It is also permeated with Eurocentric cultural values that are often implicit. Such cultural values include human control over nature, human ascendency over other life forms, separation of the neutral observer from the object of inquiry, separation of "objective" knowledge from "subjective" experience, among many others [10]. Within the context of a specialized discipline like nutrition, such ideas are easily presumed as "givens" because, as Kuhn observed, these shared understandings greatly facilitate scientific advancement [4]. For example, the search for "mechanisms of action" reflects the idea of a mechanistic universe inherited from physics and chemistry as applied to physiology and then nutrition. It subjectively projects machine-like qualities onto life forms. Mechanistic thought becomes hidden through wide acceptance; as it becomes integral to the mental models and habits of mind through which disciplinary inquiry proceeds it escapes criticism. There is no question that mechanistic thought has proved useful for advancing nutrition science as for much of biomedicine. But is it possible that over-attachment to a "living being as machine" explanatory mindset might also become a liability if it constrains our ability to recognize other relationships? I propose that employing a cultural lens will offer nutrition science an accessible means to illuminate and contemplate hidden subjectivities that are otherwise implicit within our disciplinary habits of mind. A cultural lens would serve to extend our domain of thought by offering a different mental filter than that of the current disciplinary perspective. A cultural lens could be selected and used as needed to make more visible and apparent the dimensions of scientific practice that are otherwise implicit or opaque.

Webster defines culture as "the totality of socially transmitted behavior patterns, arts, beliefs, institutions, and all other products of human work and thought typical of a population or community at a given time" [40]. This definition certainly includes the activity of producing human knowledge, including scientific knowledge. A cultural lens would help us to see scientific disciplines as not just sub-cultures in themselves, but more importantly as products of and expressions of the larger societal culture from which they emerge. Many scientific disciplines, including nutrition, tend not to see their discipline or scientific practice as an expression of European culture. Most practicing scientists seldom consider the cultural grounding of their professional training or the cultural nature of their habits of mind. Scientists rarely see their work of advancing academic knowledge as a Eurocentric cultural construction. I am sympathetic that this proposal may seem as a rather radical proposition, evoking an uncomfortable sense of dissonance. Although such dissonance can feel destabilizing, I suggest this discomfort is ultimately healthful and productive because it offers nutrition science a means to expose otherwise hidden subjectivities for peer consideration and skeptical discourse. Let us explore this dissonance a bit more deeply.

Scientific investigation is often construed as a process of standing outside of culture, of removing oneself from subjective distraction as a means of decontextualized knowing [5]. Proper scientific inquiry involves creating an ideal environment for controlled observation that is separate from undue subjective influence wherein the scientific investigator can assume a role of neutral spectator [5]. Social and cultural circumstances and interests outside the carefully constructed domain of observation and controlled experiment are often thought of as threats to objective observation and deemed irrelevant [5,6,12]. To the extent possible, gathering data and testing hypotheses in these ways are considered as either transcending culture or avoiding culture altogether. The 20^th^ century work on solving deficiency diseases offers an excellent example of how nutrition science can produce knowledge in accordance with laws considered universal - knowledge holding truth that transcends any consideration of culture. Precisely because mental models are successful, over time they become unconsciously taken for granted [3-6]. When assumptions-taken-as-truth become reified through success, there seems no need to examine what is self-evidently true. Background assumptions remain unexamined, exerting a powerful, often subconscious hold on one’s "thought style" [19]. They become integral to what Fleck describes as a structurally complete meaning system that becomes closed, circular and resistant [19]. These closed and circular dynamics cultivate invisibility that renders hidden subjectivities unavailable for criticism and skeptical discourse [3,5,6]. From within a closed system that works to avoid subjective contamination, the proposal of a cultural lens might seem absurd.

A cultural lens works to disturb the closed and circular meaning system by calling attention through critical reflection [26,27] to what is often presumed as true and universal. It exposes hidden subjectivities as cultural constructions. The pervasive subject-object dichotomy between mind and matter, between scientist and nature, between experience and reality are ideas of Eurocentric origin that saturate scientific habits of mind and mental models. When assumptions-taken-as-truth are disrupted by skeptical inquiry and illuminated as mental constructions, a sense of destabilization occurs that manifests as a dissonance, or tension of epistemology [28,30]. This destabilization can produce a profound sense of discomfort. We can choose to push away the discomfort by dismissing all of this talk as a messy and unnecessary distraction from the ongoing work of nutrition science. Or we can choose to hold the discomfort for further contemplation with the hope that something productive and useful might emerge. The latter choice can provoke us to consider the work of illuminating and questioning hidden subjectivities.

Take the ideal of objectivity. That we can point to many instances where science works should not be construed as evidence that our practice of science has met its ideal of pure objectivity, escaping any cultural influence. The ideal of skeptical inquiry would ask us to examine any such assumptions more closely. A cultural lens illuminates the ideal of the scientist as a disengaged observer as a product of European thought and culture. To what extent are we truly able to disengage from the world in striving to achieve ideals of pure objectivity and value neutrality? When, if ever, are we really meeting these ideals? To what extent and under what circumstances do we claim these ideals for our own convenience? These ideals are certainly valuable and useful, but if we truly value them we must be willing to admit that in practicing science we cannot deny our own subjective humanity. Every scientist started life as a human being long before becoming a scientist. Integrity requires us to admit when subjectivity is not filtered out by scientific methods. A cultural lens can help us begin to see how examining subjective dimensions through critically reflective inquiry may help to push nutrition science forward.

Still, this kind of work can be considered a messy and superfluous distraction to the more central task of advancing nutrition science. It can interfere with "progress" as often understood within our discipline. I agree that this work is a distraction from the ongoing, more routine disciplinary work that Kuhn refers to as "normal science" [4]. In fairness, a cultural lens would also bring some added tension, paradox and anomaly with the sometimes uncomfortable exploration of hidden subjectivities. But as the world changes, a closed system will eventually become confining and constraining, acting to limit thought and possibility [4,23,28]. In a rapidly changing world, I argue that good scientific practice would have us surface and critically reflect upon hidden subjectivities so that potential difficulties become explicit, available to peer-review and the process of self-correction. The ideal of skeptical inquiry can be considered incomplete without a critically reflective dimension of thought that can do the work of illuminating and questioning hidden subjectivities.

Nutrition science is now often presented as a true description of the material and objective reality of food and living organisms [41]. This simple descriptive stance leads to the epistemological problem of underdetermination – gaps between hypotheses and data - when background hypotheses are not articulated but presupposed as universal givens [6]. Underdetermination that remains invisible and therefore goes unexamined blinds us to significant underlying problems of epistemology. A cultural lens can allow us greater appreciation for a reality of food and living organisms that is revealed in response to our particular mental models and methods of questioning. This is where a selecting a cultural lens can offer us the added mental wavelength to make visible what is opaque in a more customary bandwidth of disciplinary thought. My argument is that we have reached a point in time where the cost/benefit equation weighs in favor of returning our attention to hidden subjectivities for the benefit of our discipline in the 21^st^ century.

I contend that adopting a cultural lens would not threaten any legitimate intellectual substance or scientific integrity, while much capacity for self-correction and possibility for innovation could be gained. A cultural lens would not negate or compromise any scientific progress or assets that a biomedical thought style and cultural orientation brings to scientific practice. Many of our hidden subjectivities are integral to success in science and to how scientific knowledge is constructed. But I suggest that protecting hidden subjectivities from the collective and rigorous inquiry that a cultural lens would bring is no longer tenable in the 21^st^ century. My hope is that proposing a cultural lens will encourage further conversation and discussion around these ideas. Perhaps at least a few scientists within the field might be predisposed to create a branch of critically reflective discourse devoted to acknowledging and examining our hidden subjectivities. This kind of discourse is now lacking in nutrition science, but adopting a cultural lens would allow it to emerge and inform the collective mindset and thought patterns of the greater discipline [28,30].

### Consequences of type II error?

As indicated earlier, the formidable authority of science to define nature and to determine what constitutes reliable knowledge of the world can condition and reify a belief among professional scientists that *legitimate knowledge can only arise* through methods accepted as valid within scientific societies. This idea certainly cannot be considered scientific; it did not result from experimental testing nor does it represent a hypothesis subjected to systematic and rigorous methodological inquiry. A cultural lens helps us to clarify this belief as an *idea about science*, an idea that becomes easily conflated with science within a structurally complete and circular meaning system [19]. This belief is certainly not without merit. It serves in defense against type I error (accepting as true what is actually false) and it is also commonly invoked as a defense against fraudulent health claims. But to what extent have we carefully considered the opportunity costs and hidden consequences that arise when "non-scientific" forms of knowledge arising from non-Eurocentric cultures are summarily dismissed or subjugated, in essence leaving the door wide open for type II error (rejecting as false what may actually be true)?

Every society in human history has had to develop its own forms of nutrition knowledge. Just as nutrition science and biomedicine are expressions of European culture, so indigenous knowledges [14,17,32,36], Ayurveda [33] and Chinese Medicine [16,34] are expressions of their cultures of origin. Attempting to understand the knowledge systems of non-European cultures exclusively through the lens of biomedical mental models is now routine practice but one that overlooks the importance and power of hidden subjectivities and cultural difference. Imposing biomedical models as a presumed universal standard for legitimation yields understandings of non-biomedical knowledge systems that are partial, distorted and fragmentary [14,16,17,28-30,36]. Presumptions of universality are yet another example of hidden subjectivity that escapes critical examination and the self-corrective power of biomedical peer-review. Such presumptions should be openly called into question and unpacked. That we can cite many instances where biomedicine works should not dissuade us from acknowledging the existence of powerful hidden subjectivities infiltrating biomedical thought styles. Because hidden subjectivities permeate human thought, we might expect that other cultures would offer their own array of background assumptions that could merit consideration on their own terms.

I was trained as a "wet bench" laboratory nutrition scientist and have subsequently worked for over twenty years as an Extension Specialist in cross-cultural contexts. My experience suggests non-biomedical knowledge systems can offer nutrition science a valuable means for critical reflection and further consideration if approached with greater appreciation for the subjective dimensions of human thought [2,29,36,37]. This possibility is foreclosed if we reflexively impose our biomedical models as the only possible means through which to gain a legitimate understanding. By regarding biomedical models as cultural constructions, not universal givens, we hold a key to a doorway of broader understanding and wider possibility. Through the doorway sits a vast intercultural field with a varied topography of human thought and cognitive terrain. Each system of knowledge is grounded within its own cultural context, its own terrain of background assumptions with its own unique standpoints. These are vantage points offering different perspectives, which when inhabited empathically allow one to see and explore food and health relationships through a different cognitive orientation [14,16,28,33,34]. A very brief armchair journey may be helpful.

It is often forgotten that, prior to being colonized by Europeans, Indigenous peoples of the Americas existed in excellent health [42]. Indigenous cultures developed sophisticated systems of agriculture that have given us beans, corn, potatoes, pumpkins, squash, tomatoes, peppers and over twenty other foods [32]. They knew how to cure scurvy centuries before Europeans [42,43]. The first Pharmacopeia of the United States published in 1820 lists more than 200 medicines coming from indigenous peoples [42,43]. Moerman reports that of the 31,566 kinds of vascular plants found in North America, American Indians used 2874 of these species as medicines, 1886 as foods, 492 as fibers for weaving, baskets, building materials etc. and 230 as dyes [43]. All told, they found a useful purpose for 3923 kinds of plants. These achievements are seldom acknowledged in nutrition science. Why?

Of course, the short answer is that the knowledge of indigenous peoples is usually considered "unscientific". We can certainly agree that indigenous knowledge is "un-Eurocentric". But is it science? Gregory Cajete answers with an emphatic "Yes" in his book "Native Science" [17]. A cultural lens would help us to understand that indigenous sciences share with biomedicine a great appreciation for keen and rigorous empiricism [14,17,36]. Most indigenous sciences do not share the extreme subject/object separation that is inherent within biomedical thought of Eurocentric origin [14,17,36]. Rather than attempt to detach oneself as an observer isolated from the natural world in order to gain more "objective" knowledge, many indigenous peoples maintain an intimate participatory relationship with an interwoven and inter-related natural world, of which food and health relationships are a prime example [14,17,36]. As we confront the idea of indigenous sciences we are confronting a realm of hidden subjectivity culturally different than that of our biomedical science.

Perhaps our ability to effectively and respectfully navigate cultural differences within this realm is significantly impaired if we are not more fully cognizant of our own hidden subjectivities. Invoking a cultural lens would not only allow us to more empathetically consider indigenous perspectives, we could use the cultural lens to ask how our ideas about subject/object separation might be serving to limit our own scope of inquiry. Consider the challenging quote from the influential Lakota scholar Vine Deloria Jr.:

*Science insists, albeit at a great price in understanding, that the observer be as detached as possible from the event he or she is observing. Indians know that human beings must participate in events, not isolate themselves from occurrences in the physical world. Indians thus obtain information from birds, animals, rivers and mountains which is inaccessible to modern science".*[44], p 40.

This quote will often evoke significant dissonance from scientists. It is important to hold this dissonance despite any feelings of destabilization. Since the time of Immanuel Kant, natural philosophy of Europe has also recognized and emphasized the participation of man’s own mind in the perception and observation of phenomena [11,35]. While this idea is largely undisputed, it is left out of account in many scientific disciplines, including nutrition, trumped by the idea that nature is to be studied *as objects independent of ourselves*. Perhaps the enormous gains in accuracy and precision derived from our lens of objectification has also excluded any residual sense of the conscious participation alluded to by indigenous scholars [14,17,36]. Perhaps there is something we can learn about ourselves from encountering and contemplating indigenous subjectivities. The late Paul Schultz, an Elder, spiritual leader and Chair of the Board of Trustees at White Earth Tribal & Community College put it this way:

*"A problem with Western Science, that is inherently its own problem, is that in its quest for excellence, in so many ways it makes the mistake of running over or not noticing what other people may have to contribute, in its effort to not only to do ‘good research’, but also to protect what scientists feel is the integrity of the scientific process".*[45]

Must concern for scientific integrity cause us to dismiss out of hand entire systems of knowledge because they seem "unscientific" upon superficial inspection? Given our overlooked and unexamined subjectivities, the powerful forces of professionalism and peer approval can lead even the most fair-minded of scientists to dismiss or hold in abeyance any human knowledge unless and until it can be tested scientifically, within the framing, models and methods deemed valid by professional peers. Invoking a cultural lens through which to view such situations allows us to culturally situate our established practices of knowledge construction. If we are able to cultivate a greater collective awareness of the contingency of our current epistemic standards, we can also begin to see other possibilities begin to emerge. Perhaps there are ways to maintain scientific integrity while being more open to the idea that culturally different knowledge systems might also have their own integrity when viewed from the standpoint of their culturally distinct background assumptions. Perhaps exploring the field of cultural difference within the realm of hidden subjectivities can help us to recognize how, epistemologically speaking, there may be more cards in the deck than we have been playing.

## Conclusion

Today’s nutrition science has its roots in Europe and its biomedical practice remains largely a Eurocentric cultural expression. It is permeated with Eurocentric values that include profound tendencies toward materialism, reductionism, mechanistic thought and pervasive subject-object dichotomies between mind and matter, between scientist and nature, between experience and reality, among many others. Owing to our detachment from philosophy and to the trajectory of scientific success, these ideas are often taken-for granted, become implicit, unavailable for peer review and overlooked in the process of disciplinary self-correction. A cultural lens revealing nutrition science as a cultural construction could have meaningful impact on our discipline in at least two important ways. First, it illuminates hidden subjectivities otherwise implicitly embedded within the ongoing practice of nutrition science. If a critical mass of nutrition scientists were to engage in this work, new avenues of discourse could offer a dimension of self-correction that is now missing within the broader collective discipline. Second, a cultural lens also makes possible a practice of cross-cultural engagement (CCE). CCE would have scientists temporarily step away from biomedical mental models to dwell within culturally different knowledge systems of food and health. This practice offers scientists different vantage points to perceive situations and think through problems. Developing a capacity over time to inhabit different cultural perspectives would open possibilities for reciprocal, intercultural inquiry with respect to other forms of cultural knowledge now often stigmatized, including Ayurveda, Chinese medicine, African and indigenous knowledges. Adopting a cultural lens offers new possibilities for disciplinary adaptation and advancement through self-correction and through engagement across cultural difference. I advocate the following actions:

1) **Require philosophy of science and history of science for students majoring in nutrition science.** These courses accommodate a cultural lens and give future professionals a larger cultural context in which to situate existing concepts and principles of nutrition science.

2) **Debate and contemplate the merits and implications of adopting a cultural lens. Create a branch of professional discourse that could inform nutrition science inquiry.** A small cadre of nutrition scientists could pursue inquiry around hidden subjectivities of the discipline as the subject of professional discourse that would inform the larger disciplinary collective.

3) **Consider adopting systems thinking and action research as complementary modes of inquiry.** Both systems thinking [24] and action research [25] can accommodate a cultural lens and explicate hidden subjectivities while complementing prevailing approaches to research. They could be considered within the branch of discourse described above.

4) **Encourage cross-cultural engagement (CCE) as a disciplinary practice.** CCE is not for everyone, but it will allow interested nutrition science professionals to learn to step into the intercultural terrain of different epistemologies and knowledge systems. Scientists can learn to more appropriately interface with diverse knowledge systems in ways that can open a greatly expanded intercultural field of possibility.

## Competing interest

The author declares that he has no competing interests.
